# Upstream open reading frames: Molecular switches in (patho)physiology

**DOI:** 10.1002/bies.201000037

**Published:** 2010-10

**Authors:** Klaus Wethmar, Jeske J Smink, Achim Leutz

**Affiliations:** 1 Max Delbrueck Center for Molecular MedicineBerlin, Germany; 2 Charité, University Medicine BerlinGermany

**Keywords:** C/EBP, mutations, translational control, tumorigenesis, uORF

## Abstract

Conserved upstream open reading frames (uORFs) are found within many eukaryotic transcripts and are known to regulate protein translation. Evidence from genetic and bioinformatic studies implicates disturbed uORF-mediated translational control in the etiology of human diseases. A genetic mouse model has recently provided proof-of-principle support for the physiological relevance of uORF-mediated translational control in mammals. The targeted disruption of the uORF initiation codon within the transcription factor CCAAT/enhancer binding protein β (C/EBPβ) gene resulted in deregulated C/EBPβ protein isoform expression, associated with defective liver regeneration and impaired osteoclast differentiation. The high prevalence of uORFs in the human transcriptome suggests that intensified search for mutations within 5′ RNA leader regions may reveal a multitude of alterations affecting uORFs, causing pathogenic deregulation of protein expression.

## Introduction

Defective translational control of protein expression is increasingly recognized as an important mechanism in the etiology of human diseases 1. In eukaryotic mRNA the main protein coding sequence (MCS) is flanked by upstream and downstream regulatory regions of variable length and structure. These regions may contain multiple regulatory cis-acting sequence elements, including 5′-located hairpins, protein binding sites, upstream open reading frames (uORFs), or internal ribosomal entry sites (IRESs) as well as 3′-located microRNA target sites, specific localization elements (zip codes) or polyadenylation signals. Several review articles have summarized how such cis-regulatory translational control elements influence translation of the MCS and how their dysfunction relates to the development of human diseases 2–7.

A growing body of evidence obtained from bioinformatic and genetic studies suggests that, in particular, uORF-mediated translational control may serve as a comprehensive mechanism of protein expression control. Recently, the targeted genetic ablation of the translational start site in the uORF of the transcription factor CCAAT/enhancer binding protein β (C/EBPβ) validated the physiological relevance of uORF-mediated translational control in an animal model 8.

This paper aims to provide a brief overview on uORF-mediated translational control in general. Moreover, we show how aberrant uORF regulation may translate into (patho)physiology, as illustrated by data obtained from analyses of C/EBP transcription factors. Finally, we outline how contemporary sequencing technologies may help to unravel the implications of uORF-mediated translational control in a multitude of as-yet-unexplained human diseases.

## uORFs – frequency, structure, and function

Translation of eukaryotic transcripts follows a coordinated sequence of events, as summarized in the ribosomal scanning model of translation 9. Initially, a 43S pre-initiation complex, consisting of the 40S ribosomal subunit, the eukaryotic initiation factor 2 (eIF2) – guanosine-5′-triphosphate (GTP) – methionyl initiator methionine-tRNA (Met-tRNA_i_^Met^) ternary complex and additional eIFs, engages with the 7-methyl-guanosine (m^7^G) mRNA cap structure located at the 5′ end of a transcript 6. The pre-initiation complex scans the mRNA toward the 3′ end until the Met-tRNA_i_^Met^ anticodon matches a functional AUG codon. Joining of the 60S ribosomal subunit completes the assembly of a fully functional ribosome and permits initiation of translation. Initially it was assumed that scanning ribosomes would generally initiate translation at the m^7^G-cap proximal AUG initiation codon 10, but subsequently an increasing number of genes were identified that differed from this “first AUG rule”. Predominantly, transcripts with long and presumably structured 5′ regulatory regions were found to frequently contain functional AUG codons upstream of the MCS (uAUGs) 11. Such uAUGs constitute the initiation codon of uORFs, and interfere with unrestrained ribosomal scanning toward the MCS initiation codon 9.

The yeast transcription factor GCN4 represents the best-studied example of uORF-mediated translational control and illustrates how uORFs can facilitate the paradoxical induction of GCN4 protein expression under conditions of reduced global translation 12, 13. The first of four uORFs within the GCN4 5′ leader is efficiently translated under both good nutritional and starvation conditions and establishes a “reinitiation mode of translation” 14 for all downstream initiation codons 12, 13. In non-stressed cells, rapid reloading of post-termination ribosomes with indispensable initiation cofactors allows immediate reinitiation at the proximal initiation sites of uORFs two to four. These uORFs exhibit specific inhibitory features, rendering the translating ribosomes incapable of reinitiating at the MCS. During amino acid starvation the availability of initiation cofactors decreases, resulting in decelerated reloading of post-termination ribosomes and leaky scanning across the inhibitory uORF start sites. A functional initiation complex can only be reassembled after prolonged progression of post-termination ribosomes, allowing the initiation at the MCS start codon and the induction of GCN4 under stress conditions. Due to their spatial and contextual organization, the four uORFs of the GCN4 transcript serve as a translational switchboard that allows the cell to rapidly respond to nutritional stress. Ultimately, the translational induction of GCN4 and the subsequent activation of GCN4 target genes adjust the cell's molecular repertoire to environmental needs.

Mechanistically, the expression of GCN4 is determined by the combined effects of leaky scanning and reinitiation events, which are sensitive to changing global translational conditions. Data obtained from the GCN4 transcript showed that the length of a uORF, the sequence context adjacent to its termination codon and the distance to the downstream initiation codon modulates the inhibitory effect of the uORF on ribosomal reinitiation 12, 13. As also confirmed for other transcripts, lengthening of the intercistronic space increases reinitiation rates from downstream AUG codons 14, while lengthening of the uORF itself results in decreased reinitiation 15. Together, these data suggest a dynamic model where initiation cofactors are stripped from ribosomes during translation of a uORF, which need to be reassembled to allow reinitiation to occur 9.

Bioinformatic surveys have now identified uORFs in 35–49% of human and rodent transcripts 2, 16, 17 and correlated the prevalence of one or multiple uORF(s) in a transcript with reduced abundance of the respective protein 18. Despite their high prevalence, uORFs are less frequent than could be expected by chance 16, and tend to be conserved among species 19, suggesting an evolutionary selection of functional uORFs. Recently, ribosomal profiling in yeast provided strong evidence for translation of uORFs *in vivo* and confirmed changing translation rates of the uORFs and the MCS of GCN4 in response to altered nutritional conditions 20.

**Figure 1:**
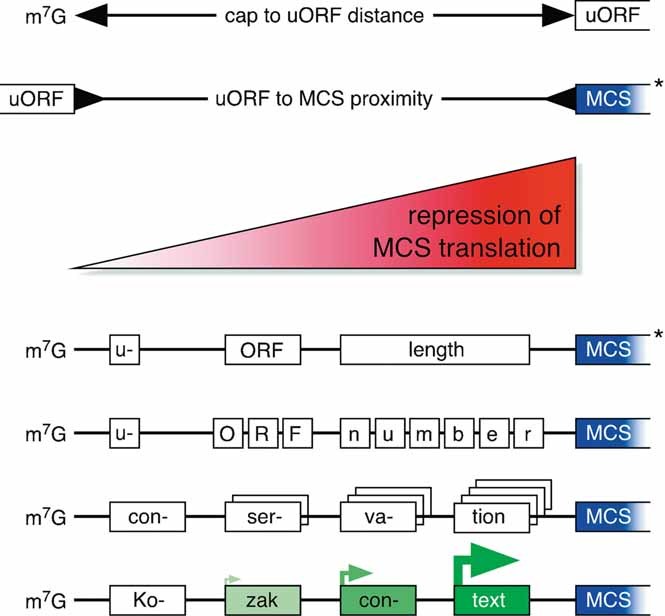
Variables affecting the degree of uORF-mediated MCS repression. The enhancement of MCS repression correlates with increasing m^7^G to uORF distance, uORF to MCS proximity, uORF length, number and conservation among species, and an increasingly favorable uORF initiation context. *These features apply to individual transcripts but have not been validated in a bioinformatic survey 15, 18, 27, 28. m7G, 5′ mRNA cap structure; uORF, upstream open reading frame; MCS, main coding sequence.

uORFs are extremely diverse in both structural features and regulatory functions. As exemplified for humans, uORFs vary in length (average of 48 nt), number per transcript (0–13), position (close to or distant from the mRNAs m^7^G-cap, terminating upstream or downstream of the MCS initiation codon), sequence (no common uORF sequence motif has been identified) and secondary structure. In approximately half of the uORF-bearing transcripts, a single uORF precedes the MCS initiation codon 18. In the remaining cases, uORF-mediated regulation is complicated by the presence of more than one uORF, and the regulatory effect on MCS translation results from the combined functions of individual uORFs, each acting in a highly context-specific manner. At present, uORF-mediated translational control has been validated experimentally for about 100 eukaryotic transcripts 18. Besides establishing barrier functions to scanning pre-initiation ribosomes, as exemplified above for GCN4, uORFs can also reduce translation of the MCS by other means. In selected transcripts, uORFs can provoke mRNA instability 17, 18 or render transcripts susceptible to nonsense-mediated mRNA decay (NMD) 21. In other cases, uORF-encoded peptides repress translation of the MCS by interaction with the translational machinery or by reducing mRNA stability in response to trans-acting molecular regulators, such as sucrose 22, arginine 23 or polyamines 24. Mass spectrometric approaches have identified a number of additional, potentially functional, uORF-encoded peptides, which await experimental examination 25, 26. Despite the overt complexity of uORF-mediated translational control, several variables correlating with strong repression of MCS translation emerge from published data. These include long 5′ cap-to-uORF distance, proximity of the uORF to the MCS initiation site, length of the uORF, multiplicity of uORFs, conservation among species, and initiation sequence context (Fig. 1) 15, 18, 27, 28.

Another intriguing regulatory function of uORFs is observed in transcripts harboring alternative downstream initiation sites within their MCS. In these transcripts, as exemplified by the transcription factors C/EBPα and C/EBPβ, uORFs control the expression ratio of functionally distinct protein isoforms by sensing the translational status of the cell 9, 29.

## How uORF regulation translates into (patho)physiology – the C/EBP paradigm

**Figure 2:**
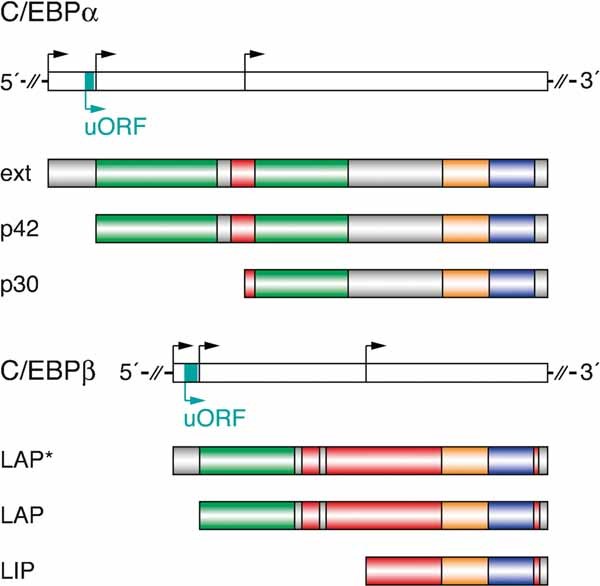
Transcripts and protein isoforms of C/EBPα and β transcription factors. Three N-terminally distinct protein isoforms (colored bars) are translated from subsequent in-frame initiation codons (black arrows) within the C/EBPα and β transcripts (open bars). Small uORFs (blue) preceding the initiation codons of C/EBPα-p42 and C/EBPβ-LAP regulate the balanced expression of long and truncated protein isoforms. The C/EBPα and β isoforms contain N-terminal trans-activating (green) and regulatory (red) domains, as well as highly conserved C-terminal basic (orange) and leucine zipper (purple) domains. The positions and sizes of indicated domains are derived from published data 32, 88–91. C/EBP, CCAAT enhancer binding protein; ext, extended; LAP, liver activating protein; LIP, liver inhibitory protein.

Evolutionarily conserved uORFs have been identified in transcripts of many key regulatory genes 30, 31, implying an important physiological role for these uORFs. Among such uORF-bearing transcripts are the transcription factors C/EBPα and β, which regulate the proliferation and differentiation of multiple cell types including granulocytes, macrophages, adipocytes, osteoclasts, osteoblasts, keratinocytes, mammary epithelial cells, and hepatocytes 32–36. C/EBP transcription factors are implicated in the regulation of various (patho)physiological processes including metabolism, inflammation, and malignant transformation 32–34, 37. C/EBPα, β, and four additional members (γ, δ, ɛ and ζ) of the C/EBP family share highly conserved C-terminal basic regions and leucine zipper domains (bZIP), which are involved in DNA binding and homo- or heterodimerization, respectively 34. The N-terminal parts of the C/EBPs are more diverse and contain regulatory and trans-activation domains that interact with transcriptional coactivators, corepressors, and the basal transcription machinery 38–40 (Fig. 2). C/EBPβ mRNA translates into two long protein isoforms known as liver activating protein (LAP) and LAP*, and the truncated isoform liver inhibitory protein (LIP). Recently, an extended C/EBPα isoform has been described 41, in addition to the known full-length p42 and truncated p30 isoforms. The full-length isoforms of C/EBPα and β both contain N-terminal trans-activating and regulatory domains that can induce differentiation and inhibit proliferation. The truncated isoforms, p30 and LIP, consist of only the C-terminal part of C/EBPα and β, respectively, retaining their DNA binding capacity and the ability to form dimers with other protein isoforms of all C/EBP family members. The absence of the N-terminal domains in p30 and LIP isoforms compromises their trans-activating functions, resulting in trans-dominant repressive effects on C/EBP target genes 42.

**Figure 3:**
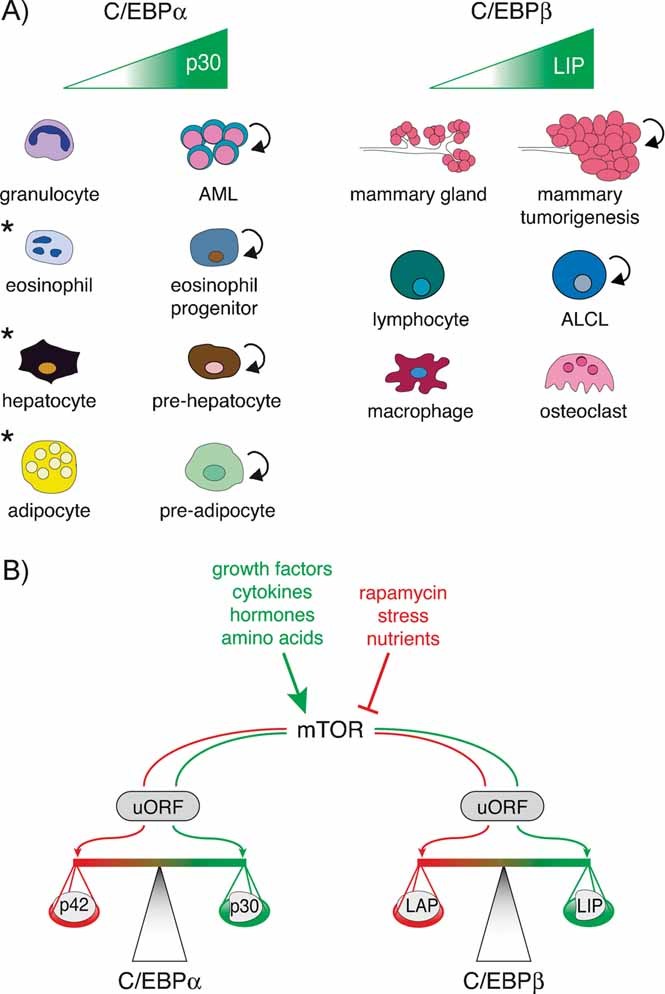
C/EBPα and β isoform expression ratios affect cellular proliferation and differentiation, and are modulated in response to mTOR signaling. **A**: Several examples of how the C/EBP isoform ratio affects cellular differentiation are illustrated, specifically how an increase in the short isoforms p30 and LIP disrupts proper differentiation. For example, p30 and LIP are overexpressed in several human cancers, including AML and breast cancer, respectively. The truncated isoforms are sufficient to induce lineage commitment of proliferative progenitor cells; however, they are not capable of blocking the cell cycle (indicated with the circular arrow) and inducing terminal differentiation and maturation. * In these cases, similar isoform specific functions have been described for both, C/EBPα and β. **B**: Environmental signals enhance (green) or repress (red) mTOR kinase activity, resulting in changes in global translational conditions. Changes in the translational status have been shown to affect uORF translation, resulting in changes in C/EBP protein isoform balance. In a good translational status, the C/EBPα and β uORFs may be more frequently translated, shifting the isoform expression ratio toward the truncated C/EBPα (p30) and β (LIP) isoforms (green).

In the transcripts of C/EBPα and β, a uORF is located out of frame between the initiation codons of the extended (α-ext and LAP*) and the full-length isoforms (p42 and LAP) 43 (Fig. 2). These uORFs were shown to be critical for the balanced expression of the respective C/EBP isoforms 29, 44. Unique and overlapping biological functions of the different C/EBPα and β protein isoforms were characterized by numerous cell biological studies. The short isoforms p30 and LIP are sufficient to induce lineage commitment of adipocytes 29, hepatocytes 45, and eosinophils 46. In addition, p30 is sufficient to commit cells to the granulocytic lineage 46, and LIP is sufficient to commit cells to the macrophage 46 and the osteoblast 47 lineages. However, the long isoforms are required to arrest the cell cycle of progenitors and to induce terminal differentiation by trans-activation of cell type-specific target genes (Fig. 3A) 29, 32, 33, 42, 45, 46, 48–53. Due to these differential effects of C/EBP isoforms in a variety of biological processes, uORF regulation was suggested to be important in determining the physiological outcome of C/EBP expression 32, 34, 37, 54.

**Figure 4:**
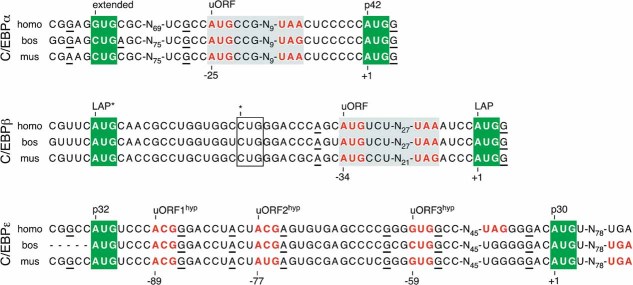
Validated and hypothetical uORFs in C/EBP transcription factors. C/EBPα and β transcripts of human (homo), cow (bos), and mouse (mus) contain experimentally validated uORFs (gray background color) terminating 7 and 4 nucleotides in front of the p42 and LAP initiation codon, respectively. The most abundant C/EBPɛ transcript variant 79 contains three subsequent hypothetical uORF initiation codons (uORF^hyp^), followed by in-frame termination codons upstream (homo) or downstream (bos and mus) of the p30 start site. The rat C/EBPɛ sequence is not displayed, as it is 100% homologous to the mouse sequence shown in this alignment. Initiation codons of protein isoforms are highlighted by green background color, initiation and termination codons of uORFs and uORFs^hyp^ are in red bold face, favorable residues of the core Kozak context (residues at −3 or +4) are underlined. * This uORF^hyp^ initiation codon may be nonfunctional, as its presence did not prevent deregulated C/EBPβ isoform expression when the uORF AUG codon (−34) was mutated to a non-functional UUG codon 8, 29.

In most tissues, C/EBPα-p42 and C/EBPβ-LAP are the most abundant protein isoforms, despite the presence of two preceding translational initiation codons and despite a suboptimal initiation codon context (Fig. 4). An optimal initiation sequence that supports initiation of virtually all scanning ribosomes is defined as CCRCC**AUG**G (Kozak consensus sequence), with a purine base in position −3 and a guanine base in position +4 as most important for initiation 55, 56. Initiation sequence contexts are frequently classified as strong (both critical residues match the consensus sequence), as adequate/intermediate (either residue −3 or +4 matches) or as weak (neither residue matches). Placing the initiation codons of the extended isoforms of C/EBPα (intermediate) and β (weak) in optimal Kozak consensus sequences resulted in loss of translation from downstream initiation codons 29, suggesting that the endogenous sequence context at the α-ext and the LAP* AUG codons allows leaky scanning, and does not support complete initiation of translation. In contrast, optimizing the Kozak context of the C/EBPα uORF start site mildly reduced translation of p42 and enhanced the expression of p30, indicating that a fraction of the post-termination ribosomes that had translated the uORF reinitiated at the proximal p42 initiation codon and another fraction initiated at the downstream p30 start site 29. The relatively high proportion of ribosomes that reinitiated at the p42 start site after translating the uORF was surprising, as the C/EBPα uORF terminates only seven bases upstream of the p42 AUG codon (Fig. 4) and intercistronic sequences of that size were known to greatly impede reinitiation rates in other transcripts 14. While strengthening of the uORF initiation sites in C/EBPα or C/EBPβ resulted in an increased p30 over p42 and LIP over LAP expression ratio, respectively, deletion of the uORF initiation codon in either C/EBPα or β enhanced expression of p42 or LAP 44 and almost completely abolished translation of the truncated isoforms 29. Therefore, the “intermediate” initiation context of p42 and LAP appeared to be sufficiently strong to support initiation of most of the scanning ribosomes in the absence, but not in the presence, of uORF translation. These observations implied that translation of the C/EBPα and β uORFs serves to shift ribosomes across the full-length initiation sites to support truncated isoform expression.

Several lines of evidence showed that the C/EBPα and β uORFs integrate the signaling status of a cell to modulate the expression ratio of isoforms. One key component in adjusting the activity of the translational machinery to environmental changes is the mammalian target of rapamycin kinase (mTOR). Many nutritional and signaling pathways downstream of growth factor-, cytokine-, or hormone receptors alter the activity of the mTOR kinase. Activated mTOR signaling is associated with enhanced global translational conditions and increased activity of important eIFs, including eIF4E 57. Mimicking favorable translational conditions by overexpression of eIF4E induced the expression of truncated C/EBP isoforms p30 and LIP (Fig. 3B) and was associated with increased initiation at the uORF start site 29. Importantly, mutation of the uORF initiation codon abolished the eIF4E-mediated induction of short C/EBP isoforms, confirming that indeed translation of the uORF was required to shift initiation toward the distal initiation codons 29. In turn, inhibition of mTOR kinase activity by the macrolide antibiotic rapamycin, protein folding stress or nutrient depletion decreased global translational activity and was associated with the predominant production of p42 and LAP isoforms 29 (Fig. 3B). In response to rapamycin treatment, a shift of expression toward the full-length C/EBPβ protein isoform was also observed for endogenous transcripts and was shown to affect cellular fates, *e.g*. the differentiation of osteoclasts 35 or the proliferation of malignant cells 58. Increased LIP over LAP isoform ratios were observed in several malignancies including Hodgkin lymphoma, anaplastic large cell lymphoma 58, and aggressive forms of breast cancer (reviewed in Ref. 37). Moreover, transgenic expression of LIP in mammary glands resulted in hyperplasia and tumorigenesis in mice, suggesting a pro-proliferative and tumorigenic potential of the LIP isoform 59. In several model systems the rapamycin-mediated inhibition of mTOR altered the isoform ratio in favor of LAP and resulted in a decrease in tumor cell proliferation 58, 60, 61.

Together, these data suggested that the uORF initiation codon may serve as a physiologically important sensor of global translational conditions, shifting the isoform expression ratio toward the truncated isoforms under favorable conditions and to the full-length isoforms under unfavorable conditions. This function may be due to the suboptimal Kozak context that surrounds the uORF initiation codon (Fig. 4), which allows the modulation of initiation rates in response to the translational status. Interestingly, although surrounded by an intermediate Kozak context as well, initiation rates at the full-length initiation codon appear to be not as sensitive to changing translational conditions. Lower variability of full-length initiation may be attributed to its location downstream of the uORF and to the fact that it is efficiently used already under steady-state conditions, but the molecular mechanisms driving the preferential use of the uORF initiation codon under favorable translational conditions remain to be identified. Despite the simplicity of a linear ribosomal scanning/reinitiation model as an explanation for uORF-mediated control of isoform expression, the translational regulation of C/EBP transcription factors might be more complex. Three-dimensional stem loop structures 62, regulatory trans-acting factors including CUGBP1 63 as well as hnRNP-microRNA interactions 64 were shown to affect C/EBP translation. Nevertheless, translation of the uORF is required to drive expression of the truncated C/EBP isoforms and represents a major determinant in the regulation of isoform expression ratios.

The recent generation of genetically altered mice, carrying a single nucleotide exchange of the ATG uORF initiation codon to TTG in the C/EBPβ gene (C/EBPβ^ΔuORF^), now confirmed the concept of uORF-mediated isoform expression *in vivo* and contributed to a deeper understanding of how changes in the isoform ratio of C/EBPβ affect mammalian physiology 8. The C/EBPβ^ΔuORF^ mice were generated using homologous recombination into the endogenous *c/ebpβ* gene locus. The ΔuORF mutation eliminates the uORF initiation codon and thus disrupts its function as molecular switch to induce the truncated LIP isoform, without alteration of the amino acid sequence of C/EBPβ. Data obtained from the C/EBPβ^ΔuORF^ homozygous mice showed that the C/EBPβ isoform production becomes unresponsive to extracellular stimuli, such as lipopolysaccharide treatment, which normally increases the LIP to LAP ratio 8. Furthermore, ablation of uORF initiation prevented the physiological induction of LIP during liver regeneration. Lack of LIP expression resulted in enhanced acute phase response, prolonged repression of cell cycle genes and impaired cell cycle entry of hepatocytes after partial hepatectomy 8. In a second recombinant mouse model (C/EBPβ^LIP^), the endogenous *c/ebpβ* gene locus was replaced by the coding sequence of the LIP isoform only, resulting in complete loss of expression of LAP* and LAP. The exclusive expression of LIP in these animals rescued both the expression of cell cycle genes and the entry of hepatocytes into S phase 8. Furthermore, C/EBPβ^LIP^ mice displayed enhanced differentiation of bone-resorbing osteoclasts, while in turn, the decreased LIP to LAP isoform ratio in C/EBPβ^ΔuORF^ mice showed an impaired osteoclast differentiation. The C/EBPβ LAP isoform was found to induce the expression of the transcription factor MafB in monocytes. MafB inhibits or sequesters other transcription factors that are known to mediate osteoclastic differentiation, including Fos, Nfatc1, and Mitf. In contrast, LIP downregulates MafB expression, resulting in increased availability of those osteoclastic transcription factors 8, 35, 65. In summary, the C/EBPβ^ΔuORF^ mouse comprises the first genetic animal model that confirms the physiological significance of uORF translation *in vivo* and its action as a molecular switch driving cell fate decisions by modulating isoform expression ratios. The *in vivo* data support the idea that the abundance of individual C/EBPβ isoforms is regulated by uORF-mediated integration of cellular signals, resulting in tissue-specific functions that depend on the cellular context.

These and other data also challenge the model of LIP being a general transcriptional repressor and of LAP*/LAP acting as general trans-activators. LIP has now been described as a trans-activator in several situations, *e.g*. on target genes containing C/EBP-responsive promoter elements that can be mutually activated by LIP or cyclin D1 66, as well as in osteoblasts by interaction with the osteoblastic transcription factor Runx2 47. The trans-activation potential of LIP might be explained by LIP out-competing the repressive effects of long C/EBP isoforms, as described for E2F target genes 8, 67, 68. Moreover, LIP may enhance differentiation of osteoblasts and osteoclasts 35. These observations suggest a high versatility and target gene specificity in C/EBPβ isoform functions.

For C/EBPα, data obtained from patients and targeted mouse genetics also argue for critical physiological functions of the C/EBPα uORF in balanced isoform expression. C/EBPα is an inducer of terminal differentiation in granulocytes and couples induction of cell type-specific genes to cell cycle arrest 32, 33, 69. The C/EBPα full-length isoform p42, similar to the long isoforms of C/EBPβ, blocks cell cycle progression by repressing E2F target genes, a function that is required in terminal cellular differentiation. In contrast, the truncated p30 isoform is not capable of repressing E2F target genes, and therefore proliferation continues, preventing terminal differentiation 70–72. C/EBPα is mutated in about 10% of patients with acute myeloid leukemia (AML), where the most common mutations result in the loss of p42 expression, while the production of p30 is preserved 73–76. A myeloid proliferative phenotype due to loss of p42 expression was also observed in knock-in mice that express p30 only 77. These mice displayed disturbed granulopoiesis and premature death. Presence of p30 was sufficient for progenitor commitment to the granulocyte-macrophage cell lineage; however, p42 was required to restrain proliferation of these myeloid progenitors, and its absence resulted in a myeloid proliferative disease resembling human AML 77. Furthermore, pharmacologically induced differentiation of AML cells by the triterpenoid 2-cyano-3,12-dioxooleana-1,9-dien-28-oic acid (CDDO) required an intact uORF 78. Underlining the critical importance of the C/EBPα uORF, a null mutation of its initiation codon in mice results in early embryonic lethality (A. Bremer and C. F. Calkhoven, personal communication).

Another example of how isoform expression ratios affect cell fate decisions comes from C/EBPɛ, the third C/EBP family member that is produced as various N-terminally truncated isoforms. The C/EBPɛ gene differs structurally from C/EBPα and β in that it contains introns. In addition to alternative translational initiation, the expression of four alternative C/EBPɛ isoforms (p32, p30, p27, and p14) was attributed to differential promoter usage and alternative splicing. Similar to the short C/EBPα and β isoforms, the short C/EBPɛ isoforms display less trans-activation potential, with the shortest isoform (p14) virtually lacking trans-activating domains 79–81. C/EBPɛ is expressed in hematopoietic cells of the granulocytic lineage, and is required for the terminal differentiation of granulocytes into eosinophils and neutrophils 79, 80, 82. Recent studies showed that the isoforms of C/EBPɛ differentially affect granulocytic lineage commitment and differentiation pathways 81. Despite many structural and functional similarities between C/EBPα, β, and ɛ, it remains to be determined whether uORF-mediated translational control also affects C/EBPɛ isoform expression. Only the murine and rat transcripts contain an out-of-frame uAUG codon between the p32 and the p30 translational start site (Fig. 4). Nevertheless, as many as three conserved hypothetical C/EBPɛ uORFs could initiate from alternative out-of-frame initiation codons in the human transcript, such as ACG at −89 and −77 (which corresponds to the mouse and rat uAUG) or GUG at −59 in respect to the adenine base in the p30 initiation codon (Fig. 4). All three hypothetical uORF (uORF^hyp^) start sites are conserved, but the uORF^hyp^ terminates five bases upstream of the p30 initiation codon only in humans, while in cow and mouse it overlaps the p30-coding sequence by 85 nucleotides. Given that all three potential uORF start sites are surrounded by intermediate or favorable Kozak consensus sequences, uORF-mediated translational control might be an additional level of C/EBPɛ expression regulation.

## Mutant uORFs accounting for human diseases

In analogy to the experimentally deleted C/EBPβ uORF initiation codon in C/EBPβ^ΔuORF^ mice, naturally occurring uORF mutations in other genes may cause physiological alterations by deregulating translation of the affected transcript. Such mutations could either change the presence of a uORF by generating or deleting an initiation codon upstream of the MCS start site, or could affect translational control by changing one of the structural features of an existing uORF (Fig. 1). More than 500 single nucleotide polymorphisms (SNPs) have been identified in humans that either create or delete uORFs, highlighting the potential physiological implications of uORF-mediated translational control. This variability in the presence of uORFs may suggest a substantial contribution of uORF-mediated regulation to individual phenotypes and/or the predisposition to distinct diseases 18. To date, three well-documented and thoroughly analyzed uORF-affecting mutations have been linked to the development of human diseases: (i) hereditary thrombocythemia is caused by a mutation that eliminates a uORF due to the generation of an alternatively spliced mRNA, resulting in increased production of thrombopoietin protein 83; (ii) reduced production of cyclin-dependent kinase inhibitor 2A, caused by a mutation that introduces a uORF in the 5′ leader sequence of the CDKN2A transcript, results in familial predisposition to melanoma development 84; and (iii) Marie Unna hereditary hair loss is caused by variable mutations altering a uORF within the hairless homolog (HR) transcript, causing an increased expression of the hairless homolog protein 85. This list was recently extended by 11 disease-related genes, where uORF-altering mutations were identified by computational analysis of the Human Gene Mutation Database 18. Diseases with confirmed uORF mutations include the van der Woude syndrome (IRF6), hereditary pancreatitis (SPINK1), and familial hypercholesterolemia (LDLR) 18. Additionally, the expression of the beta secretase BACE1, related to Alzheimer's disease 86, or the transmembrane receptor tyrosin kinase ERBB2, related to breast cancer 87, is at least partially controlled by uORFs. Whether deregulated uORF-mediated translational control is the crucial pathogenic event in these latter cases remains to be established. Even with only a few unequivocal cases at this time, it is evident that uORF mutations may be involved in a wide variety of diseases including malignancies, metabolic or neurologic disorders, and inherited syndromes. As many important regulatory proteins, including cell surface receptors, tyrosine kinases, and transcription factors act in a dose-dependent fashion, uORF mutations that affect expression levels of these genes might be responsible for a number of as-yet-unexplained pathologies.

## Conclusions and prospects

**Figure 5:**
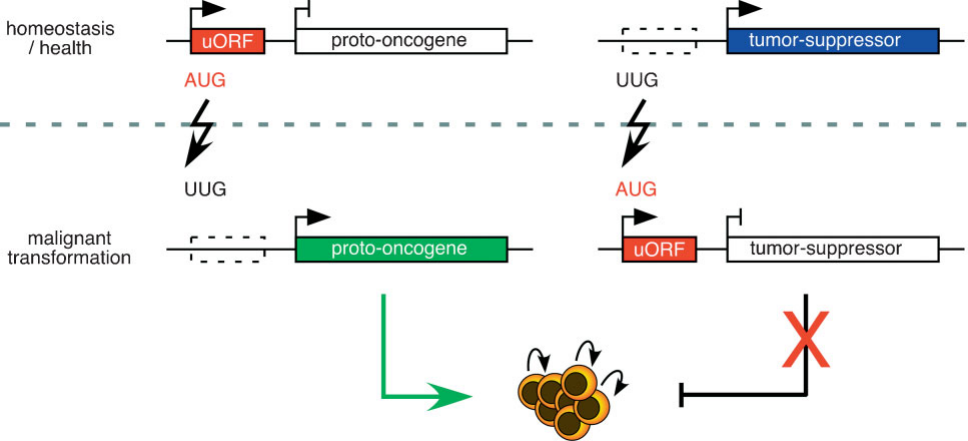
How uORF mutations may drive malignant transformation. Mutations (lightning arrows) that eliminate uORFs may activate the translation of transforming proto-oncogenes. Mutations that create uORFs in front of tumor suppressor genes may decrease translation of the encoded protective protein (as shown for CDKN2A 84). Either way, uORF-affecting mutations may result in malignant transformation of cells.

The recent validation of the (patho)physiological importance of uORF translation in mice added a new level of significance to this cis-regulatory mechanism of translational control. C/EBPα and β transcription factors represent well-established examples of how translational control by uORFs may affect cell fate decisions. Accumulating evidence suggests that deregulated uORF function might be a widespread mechanism underlying the development of human diseases. The rapid progress in advanced sequencing technologies will permit screening approaches to identify causative uORF mutations in primary material derived from patients. Malignancies of the blood might be among the most suitable types of diseases to start such an analysis, as cell samples are readily accessible. One would, *e.g*. expect to uncover mutations resulting in a “loss of uORF function” in proto-oncogenes, causing their ectopic and transformation-inducing overexpression. In turn, mutations yielding a “gain of uORF function” in tumor suppressor genes may result in malignant transformation due to a decreased production of protective proteins (Fig. 5). Given the high number of human transcripts carrying at least one uORF, the in-depth analysis of 5′ leader sequence mutations has the potential to substantially widen the spectrum of diseases with molecularly resolved etiology. Uncovering disease-related uORF mutations will inspire extensive subsequent research aiming to target the misexpressed proteins for therapeutic intervention.

## Abbreviations

AML: acute myeloid leukemia
CDDO: 2-cyano-3,12-dioxooleana-1,9-dien-28-oic acid
C/EBP: CCAAT/enhancer binding protein
eIF: eukaryotic initiation factor
LAP: liver activating protein
LIP: liver inhibitory protein
MCS: main protein coding sequence
Met-tRNA_i_^Met^: methionyl initiator methionine-tRNA
m^7^G: 7-methyl-guanosine
mTOR: mammalian target of rapamycin kinase
NMD: nonsense-mediated mRNA decay
SNP: single nucleotide polymorphism
uORF: upstream open reading frame
